# Differences in the tissue tropism to chicken oviduct epithelial cells between avian coronavirus IBV strains QX and B1648 are not related to the sialic acid binding properties of their spike proteins

**DOI:** 10.1186/1297-9716-45-67

**Published:** 2014-06-14

**Authors:** Ann-Kathrin Mork, Martina Hesse, Sahar Abd El Rahman, Silke Rautenschlein, Georg Herrler, Christine Winter

**Affiliations:** 1 Institute of Virology, University of Veterinary Medicine Hannover, Bünteweg 17, 30559 Hannover, Germany; 2 Department of Virology, Faculty of Veterinary Medicine Mansoura University, Mansoura, Egypt; 3 Clinic for Poultry, University of Veterinary Medicine Hannover, Bünteweg 17, 30559 Hannover, Germany

## Abstract

The avian coronavirus (AvCoV) infectious bronchitis virus (IBV) is a major poultry pathogen. A characteristic feature of IBV is the occurrence of many different strains belonging to different serotypes, which makes a complete control of the disease by vaccinations a challenging task. Reasons for differences in the tissue tropism and pathogenicity between IBV strains, e.g. a predilection for the kidneys or the oviduct are still an open question. Strains of the QX genotype have been major pathogens in poultry flocks in Asia, Europe and other parts of the world. They are the cause of severe problems with kidney disease and reproductive tract disorders. We analysed infectivity and binding properties of the QX strain and compared them with those of the nephropathogenic strain B1648. As most IBV strains do not infect permanent cell lines and show infection only in primary chicken cells of the target organs, we developed a culture system for chicken oviduct explants. The epithelial cells of the oviduct showed a high susceptibility to infection by the QX strain and were almost resistant to infection by the nephropathogenic B1648 strain. Binding tests with isolated primary oviduct epithelial cells and soluble S1 proteins revealed that S1 proteins of two IBV strains bound with the same efficiency to oviduct epithelial cells. This attachment was sialic acid dependent, indicating that the sugar binding property of IBV spike proteins is not the limiting factor for differences in infection efficiency for the oviduct of the corresponding viruses.

## Introduction

Coronaviruses are pathogens of birds and mammals including humans. The avian coronavirus (AvCoV) infectious bronchitis virus (IBV) as a representative of the *Gamma-coronavirus* genus infects mainly chickens and other galliforme birds. Within the species AvCoV, there are many strains belonging to different serotypes, genotypes and/or different pathotypes. Some of these strains cause only respiratory disease whereas other strains can spread to other organs like the kidneys and the reproductive tract (reviewed in [1]). The clinical manifestations of IBV in the kidney and the oviduct are of high economic importance in the poultry business. When the kidneys of young broilers are affected, mortality rates may be as high as 60% [2]. An infection of the reproductive tract may have severe implications comprising a drop in egg production, bad egg quality and the occurrence of so-called false layers. Viruses of the QX genotype have been related to problems in layer flocks causing cystic oviducts and inducing false layers [3,4]. The chicken oviduct is a large organ, where secretion of egg white and the egg shell development takes place. It is divided into four different functional parts, including the infundibulum, the magnum, the isthmus and the uterus. In all four parts, a sheet of epithelial cells forms the outer cell layer facing the lumen of the oviduct. Whether epithelial cells of the different segments of the oviduct differ in their susceptibility to infection by IBV or whether IBV strains differ in the ability to infect oviduct epithelial cells is not known. Recent publications discuss a high nephropathogenic potential of the QX strain [5], indicating that this strain has a broad tissue tropism in the bird, which may- at least in part- explain the high pathogenicity. The B1648 strain has a predilection for the kidneys and has been shown to reproducibly induce kidney disease [6]. An involvement of the reproductive organs in a B1648 infection has not been described.

It has been reported that differences in the organ and cell tropism and thus differences in the pathogenicity of IBV strains may be associated with differences in the binding properties of their spike proteins [7,8]. As the binding to susceptible host cells is the first important step in a virus life cycle, this would partly explain why some strains are able to spread to kidneys and/or to the oviduct of chickens. For several coronaviruses, the receptors have been identified. For IBV no cellular protein is known to function as a cellular receptor, but alpha 2,3-linked sialic acids serve as receptor determinants for this virus [9]. The importance of sialic acids for the infection of chicken host cells was shown for both, the QX strain and the B1648 strain [10,11]. We analysed in this study whether the differences in the ability of the two viruses to infect oviduct epithelial cells were related to differences in binding properties of their spike proteins.

## Materials and methods

### Viruses

Virus stocks of the IBV strains QX and B1648 were obtained by propagation in specific pathogen free embryonated chicken eggs (VALO SPF, Cuxhaven, Germany). The allantoic fluid was harvested, clarified by low–speed centrifugation and stored at −80 °C. The viral titer was determined by titration in primary chicken embryo kidney cells. The IBV strain QX was kindly provided by Hans Christian Philipp (Lohmann Tierzucht, Cuxhaven, Germany). The IBV strain B1648 was kindly provided by Dave Cavanagh (Institute for Animal Health, Compton, UK). S1 sequences can be found in Additional file 1.

### Cells

Primary chicken embryo kidney cells were prepared from 20 day old SPF chicken embryos as described previously [9].

Epithelial cells of the chicken oviduct from 16–19 weeks old chickens were isolated by opening the magnum segment of the oviduct longitudinally and cutting it into small pieces. After incubation with 0.4 mg protease from *Streptomyces griseus,* Type XIV (Sigma-Aldrich, Steinheim, Germany)/mL medium for 3 h, the remaining tissue was removed and the cells in the supernatant were pelleted by centrifugation. The pellet was resuspended with Dulbecco’s modified Eagle medium and Ham’s F12 1:1, supplemented with 5% fetal bovine serum (Biochrom, Berlin, Germany), 1% chicken serum (Sigma-Aldrich, Steinheim, Germany), 1% nonessential amino acids (PAA, Pasching, Austria), 1% Penicillin/Streptomycin (PAA, Pasching, Austria), 0.01% Gentamycin (PAA, Pasching, Austria), 0.01% Amphothericine B (Sigma-Aldrich, Germany), filtered through 100 μm cell strainer and seeded in tissue culture flasks (75 cm^2^). After incubation for 2 h the cells were seeded in 6-well-plates and incubated at 37 °C and 5% CO_2_.

### Preparation of oviduct explants

The oviduct was aseptically collected from 16 to 18 weeks old premature SPF-chicken. After washing the oviduct with phosphate-buffered saline (PBS), the different segments of the oviduct (infundibulum, magnum, isthmus, uterus) were cut into approximately 5 mm thick rings and incubated at 37 °C For cultivation we used the same medium mentioned above. The next day the explants were used for infection studies with the strains QX and B1648. All experiments were performed in accordance with German animal welfare regulations.

### Viability of oviduct explants

In a live and dead staining (LIVE/DEAD Viability/Cytotoxicity Kit, Invitrogen, Darmstadt, Germany) we tested the oviduct rings for viability. Living cells are stained in green, dead cells are stained in red. Additionally we analyzed the oviduct rings for ciliary activity under the light microscope.

### Preparations of cryosections of infected oviduct explants

The explanted oviduct rings were infected 24 h post preparation with the IBV strains IBV QX and B1648 at a titer of 1 × 10^4^ foci-forming units (FFU)/ring in duplicates in each of in total three experiments. The infected rings were incubated for 1 h at 37 °C on a shaker. After 12 h, 24 h and 48 h the rings were mounted on filter papers with tissue freezing medium (Jung, Heidelberg, Germany) and were subsequently frozen in liquid nitrogen and stored at −80 °C until they were sectioned (10 μm) with a cryostat (Reichert-Jung, Nußloch, Germany).

### Immunofluorescence analysis

The cryosections were fixed with 3% paraformaldehyde for 20 min and subsequently permeabilized with 0.2% Triton-X-100 followed by three washing steps with PBS. To detect infected cells, a monoclonal antibody Ch/IBV 48.4 (Prionics, Lelystad, the Netherlands) directed against the N protein of IBV in a dilution of 1:100 was used. Bound antibodies were visualized by FITC-labeled anti-mouse antibodies (Sigma-Aldrich, Steinheim, Germany). All antibodies were diluted in 1% bovine serum albumin (Roth, Karlsruhe, Germany) and incubated with the sections for 1 h at room temperature in a humid incubation chamber. Fluorescence microscopy was performed with a Nikon Eclipse T*i* Microscope.

### Neuraminidase treatment of oviduct explants

Explants of the oviduct had been prepared as described above and 24 h post preparation the explants were treated with each 300 mU neuraminidase from *Clostridium perfringens* (Sigma-Aldrich, Steinheim, Germany) for 1 h at 37 °C. After careful washing of the explants they were infected with 1 × 10^4^ FFU/explant of IBV QX. 24 hours post infection (hpi) the explants were snap frozen in liquid nitrogen and were used for cryosection preparation.

### Lectin staining of oviduct epithelial cells

To detect alpha 2,3-linked sialic acids we used the lectins *Maackia amurensis* agglutinin (MAA) in its two isoforms MAA I and MAA II (Vector Laboratories, USA). The MAA I lectin was FITC labeled. To detect alpha 2,6-linked sialic acids we used the lectin *Sambucus nigra* agglutinin (SNA) labeled with FITC (Vector Laboratories, USA). Cryosections of the different oviduct parts were incubated with the indicated lectins for 1 h at room temperature. MAA II was used after preincubation of sections with an Avidin/Biotin Blocking kit (from Vector Laboratories, USA). Detection of bound MAA II lectins was carried out with streptavidin labeled with Cy3 (Sigma-Aldrich).

### Preparation of soluble spike proteins

cDNA of the S1 subunits was generated by RT-PCR of the indicated strains and cloned into the vector pCG1_Fc to obtain fusion proteins consisting of the S1 gene part (AA 1–536 for B1648 and AA 1–524 for QX) fused to the human IgG Fc domain. For the production of soluble spike proteins, BHK 21 cells were transfected using polyethylenimine (Polysciences, Eppelheim, Germany) with the plasmids following the instructions of the manufacturer. Supernatants containing the soluble proteins were harvested 24 h and 48 h post transfection.

To concentrate the proteins in the supernatant, *Amicon Ultra-15 Centrifuge Filter* devices were used. Aliquots of the soluble proteins were stored at −20 °C.

### Binding assay on cryosections of the oviduct

To analyze binding of the soluble spike proteins of the strains QX and B1648, we incubated the soluble proteins on cryosections of the oviduct at 4 °C for 1 h. To test if binding is sialic acid dependent we incubated the cryosections with 300 mU neuraminidase from *Clostridium perfringens* (Sigma-Aldrich, Steinheim, Germany) for 1 h at 37 °C before the incubation with the soluble proteins. Binding was detected with a secondary anti-human-Fc Cy-3 labeled antibody in a concentration of 1:750 (Sigma-Aldrich, Steinheim, Germany).

### Binding of virus particles

Allantoic fluid containing 3 × 10^5^ FFU of either QX or B1648 and 3 mL allantoic fluid of uninfected SPF chicken eggs was ultracentrifuged through a 25% sucrose cushion at 100 000 × *g* for 2 h at 4 °C. The pelleted virus particles were resuspended in PBS and applied to cryosections of the isthmus part of the chicken oviduct for 1 h at room temperature. After three washing steps a monoclonal antibody directed against the spike protein (Prionics, Lelystad, the Netherlands) and followed by a FITC labeled secondary antibody was used to detect bound virus particles.

### Flow cytometric analysis of isolated oviduct epithelial cells with bound spike proteins

To quantify the binding of soluble S1 proteins to isolated chicken oviduct epithelial cells, the isolated cells were seeded in 6-well plates (Greiner, Frickenhausen, Germany). The next day the cells were washed with PBS and detached using 400 μL trypsin and the cells were subsequently resuspended in PBS containing 1.0% bovine serum albumin. After pelleting by centrifugation at 1000 rpm for 5 min, the cells were incubated with the soluble S1 proteins for 1 h at 4 °C in an overhead shaker. After 3 washing steps with PBS containing 1.0% BSA and pelleting of the cells by centrifugation at 1000 rpm for 5 min after every washing step, the cells were incubated with an anti-human PE-conjugated secondary antibody in a concentration of 1:200 (Beckman-Coulter, Marseille, France) for 1 h at 4 °C in an overhead shaker. After three more washing steps with PBS containing 1.0% BSA, the cells were directly subjected to flow cytometric analysis on a Beckman Coulter Epics XL flow cytometer and analyzed using EXPO32 analysis software.

## Results

### Epithelial cells of the oviduct are highly susceptible to the QX strain

**Figure 1:**
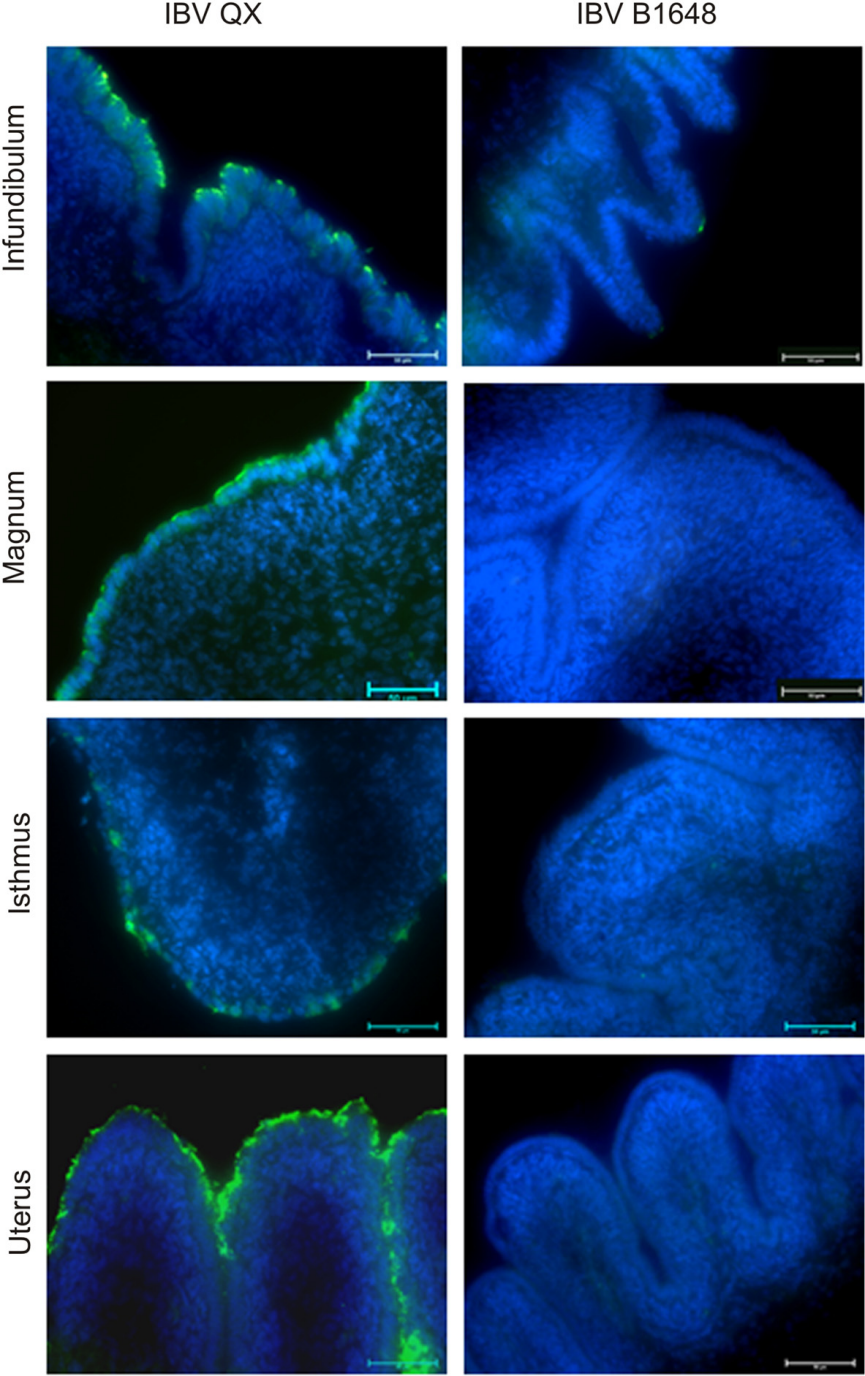
**Infection of oviduct explants with IBV strains.** The infundibulum, magnum, isthmus and uterus were infected with IBV QX and IBV B1648. Rings were fixed at 12 hpi. The nuclei were stained with DAPI (blue). The infection was detected with a monoclonal antibody against the N-protein and a FITC-labeled secondary antibody (green). Scale bar = 50 μm.

Explant cultures of premature oviducts were prepared and analysed for their viability with a live/dead staining kit. The majority of the epithelial cells were alive for more than 72 h (data not shown). An advantage of the preparation of this oviduct explants is that the epithelial cells are in the same arrangement as in vivo. The oviduct explants of the different segments infundibulum, magnum, isthmus, and uterus were infected 24 hours post-preparation with 10^4^ FFU of either of two IBV strains, QX and B1648. At 12, 24 and 48 hpi the organ explants were frozen in liquid nitrogen and cryosections were prepared and stained for IBV antigen. We observed clear differences between the IBV strains. The epithelial cells of all segments were highly susceptible to infection with the QX strain, which led to a high antigen detection rate and an increasing cytopathogenic effect over the three analysed time points. Infection with the B1648 strain was only detected in the infundibulum at very low rates. (Figure 1 and Table 1).

**Table 1:** Infection of chicken oviduct explants with the IBV strains QX and B1648

	**IBV QX 12 hpi**	**IBV B1648 12 hpi**	**IBV QX 24 hpi**	**IBV B1648 24 hpi**	**IBV QX 48 hpi**	**IBV B1648 48 hpi**
**Infundibulum**	**+++**	**+**	**+++**	**+**	**++**	**-**
**Magnum**	**+++**	**-**	**+++**	**-**	**++**	**-**
**Isthmus**	**++**	**-**	**+**	**-**	**+**	**-**
**Uterus**	**+++**	**-**	**++**	**-**	**+**	**-**

Infection score: − no infection detectable, + infection of 1–10 cells, ++ infection of 11–25 cells, +++ infection of 26 and more cells per cryo-section.

### Infection of oviduct epithelial cells with QX is sialic acid dependent

**Figure 2:**
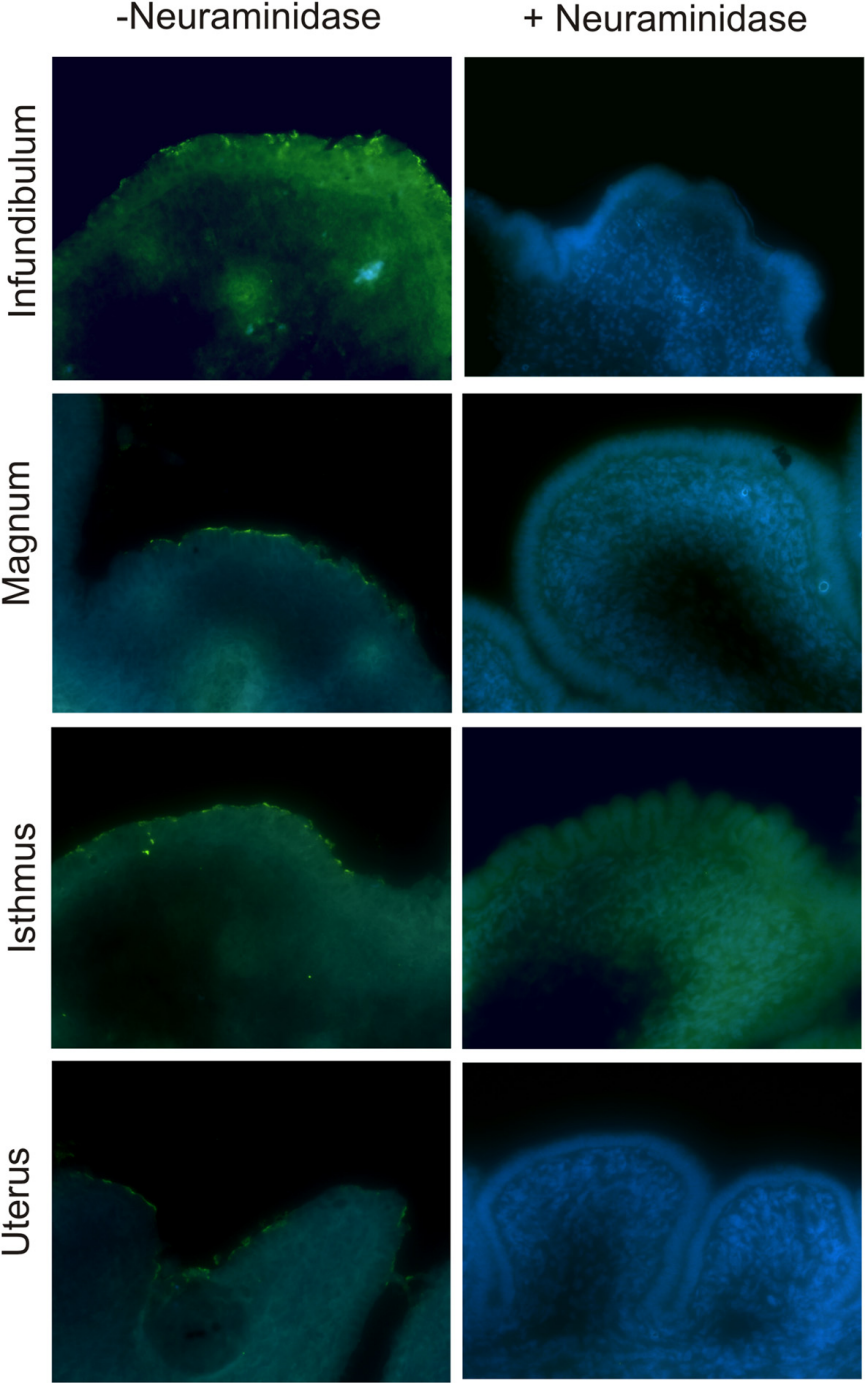
**Sialic acid dependent infection of oviduct explants with the QX strain.** Explants were treated with neuraminidase to remove the sialic acids. After infection with the QX strain 12 hpi antigen could only be detected in mock treated explants. The nuclei were stained with DAPI (blue). T the infection was detected with a monoclonal antibody against the N-protein and a FITC-labeled secondary antibody (green).

To analyse the importance of sialic acids for the infection of oviduct epithelial cells by IBV, explant cultures of the oviduct were mock-treated or treated with neuraminidase to release sialic acids from the cell surfaces. Desialylated and control samples were infected with 10^4^ FFU of the QX strain. At 12 hpi the explants were frozen and cryosections were prepared and stained for the presence of IBV antigen. After neuraminidase treatment of the explants hardly any infected cells were detectable, whereas in the mock treated explants the majority of the epithelial cells in all sections of the oviduct were infected (Figure 2). This result indicates that the infection of oviduct epithelial cells by the QX strain requires the presence of sialic acids on the cell surface.

### Lectin staining revealed expression of alpha 2,3 linked sialic acids in all parts of the chicken oviduct

**Figure 3:**
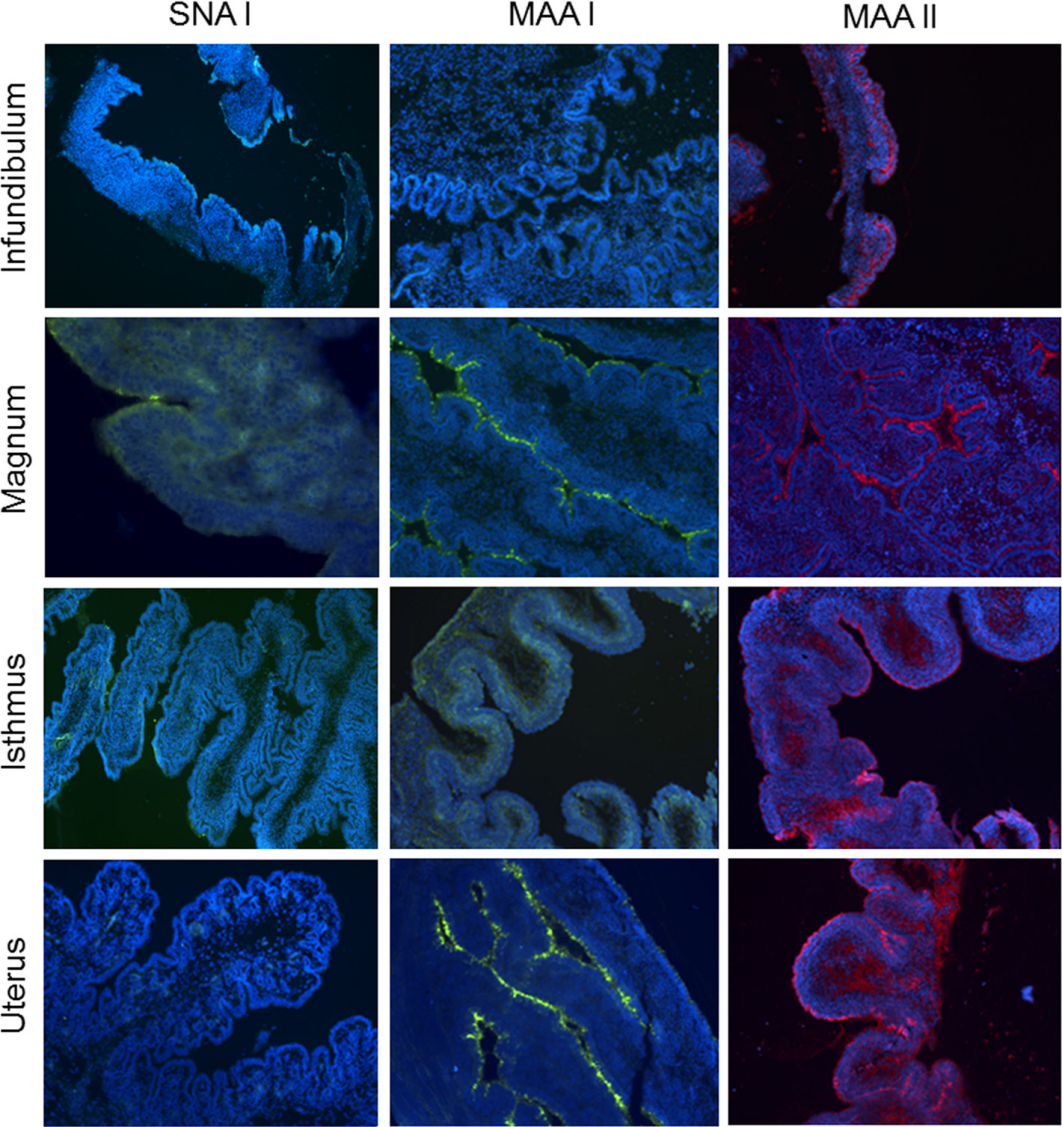
**Lectin binding on oviduct explants.** Lectin staining of oviduct explants with *Sambucus nigra agglutinin* (SNA) to detect the expression of alpha 2,6-linked sialic acids *and Maackia amurensis agglutinin* (MAA) in its two isoforms MAA I and MAA II to detect the expression of alpha 2,3-linked sialic acids. Results are summarized in Table 2.

To analyze if the receptor determinant of IBV, alpha 2,3 linked sialic acid [9,11], is expressed in all parts of the oviduct or whether variations in the expression of this sugar may explain the observed differences in the susceptibility of the cells to IBV infections we performed lectin staining of cryosections of oviduct explants. We applied two isoforms of the lectin *Maackia Amurensis* agglutinin (MAAI and MAAII) to detect alpha 2,3-linked sialic acid as well as *Sambucas nigra* agglutinin (SNA) to detect alpha 2,6-linked sialic acids. We observed that MAAII bound in all parts of the oviduct indicating expression of alpha 2,3 linked sialic acids on the majority of epithelial cells, whereas the isoform MAAI bound only in the magnum and uterus. A staining with SNA revealed that alpha 2,6 linked sialic acids were present on the epithelial cells of infundibulum, magnum and isthmus, and absent on cells of the uterus (Figure 3 and Table 2).

**Table 2:** Lectin binding on oviduct explants

	**SNA**	**MAA I**	**MAA II**
**Infundibulum**	**++**	**-**	**+++**
**Magnum**	**+**	**++**	**++**
**Isthmus**	**+**	**-**	**+++**
**Uterus**	**-**	**+++**	**+++**

Intensity of binding: − no binding, + weak binding, ++ intermediate binding, +++ strong binding.

### Soluble S1 proteins of QX and B1648 bind both to oviduct epithelial cells and in a sialic acid dependent manner

**Figure 4:**
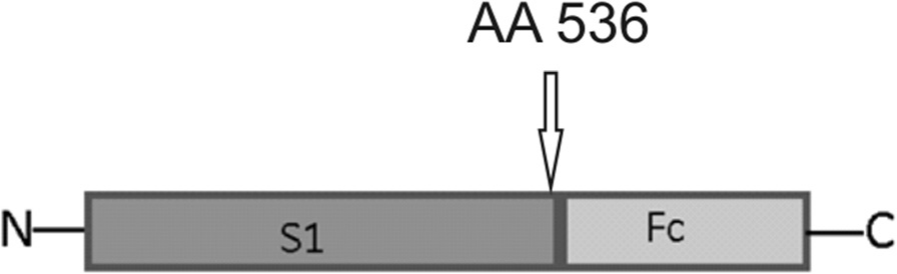
**Schematic drawing of a soluble S1 protein construct.** It consists of the S1-unit (aminoacids 1–536 of the spike proteins) of IBV QX- or B1648 spike protein fused to a human IgG-Fc tag.

**Figure 5:**
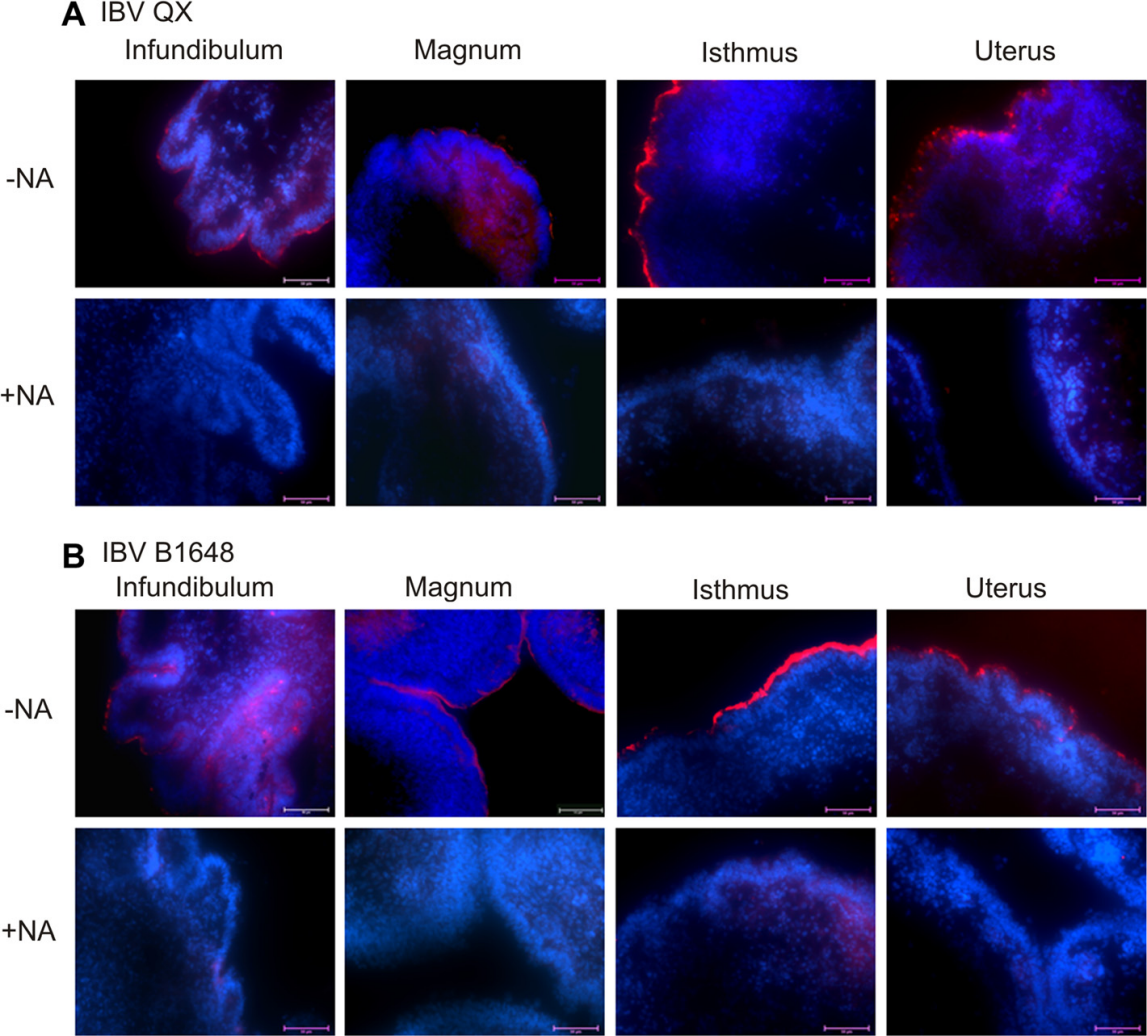
**Cryo-sections of indicated parts of the oviduct incubated with soluble S1 proteins of the IBV strains QX (A) and B1648 (B).** The nuclei were stained with DAPI (blue), binding of the soluble proteins was detected with a Cy3-labeled secondary antibody (red) at the apical side of the epithelium. To test if binding is sialic acid dependent, each approach was performed either with a pretreatment of 300 mU Neuraminidase (+NA) section or without the pretreatment (−NA). Scale bar = 50 μm.

**Figure 6:**
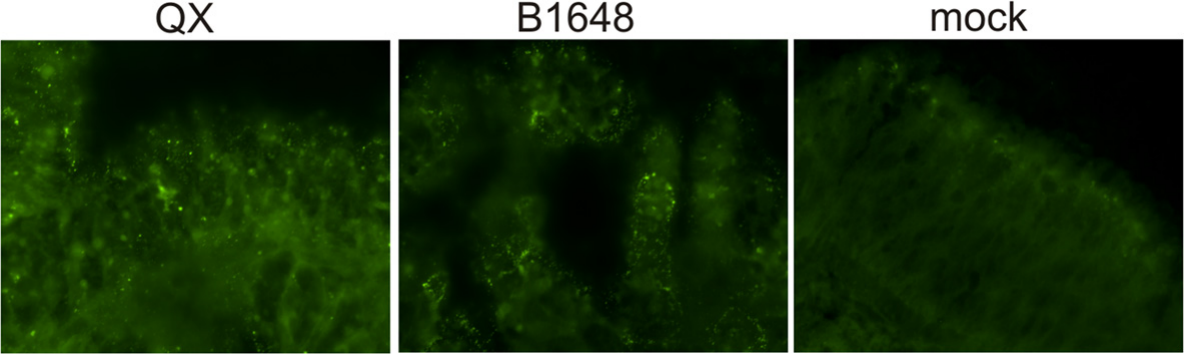
**Binding of virus particles to oviduct epithelial cells.** Viral particles of QX and B1648 strains, obtained by ultracentrifugation of virus containing allantoic fluids through a 25% sucrose cushion, were both able to bind to the epithelial cells of the oviduct. Binding was detected with a monoclonal antibody directed against the spike protein. Pictures were made using a 100 fold oil immersion objective.

To find out whether the different parts of the oviduct differ in the presence of binding sites for IBV, binding tests were performed with S1 protein connected to the Fc portion of human IgG (Figure 4). The S1 subunit is sufficient for attachment to host cells. Cryosections of the different segments of the oviduct were incubated with the S1-Fc proteins. Binding of S1-Fc was detected on all four parts of the oviduct, but staining was most intense in the isthmus whereas in the uterus fluorescent signals were restricted to single cells. This variation was observed irrespective of the source of the S1-protein, i.e. S1-Fc from strain QX showed a similar binding profile compared to the S1 from B1648. Binding of both proteins was dependent on the presence of sialic acid on the surface of the oviduct epithelium because detection of fluorescent signals was largely abolished after neuraminidase treatment (Figure 5). To analyse whether the observed binding of the soluble S1 proteins is comparable to that of trimerized spike proteins of virus particles, we performed a binding test with virions on oviduct epithelial cells. Both, QX and B1648 viruses could bind to epithelial cells, indicating that the soluble spike constructs have similar binding properties as the viruses (Figure 6).

### No differences of the binding intensity of S1 proteins to oviduct epithelial cells

**Figure 7:**
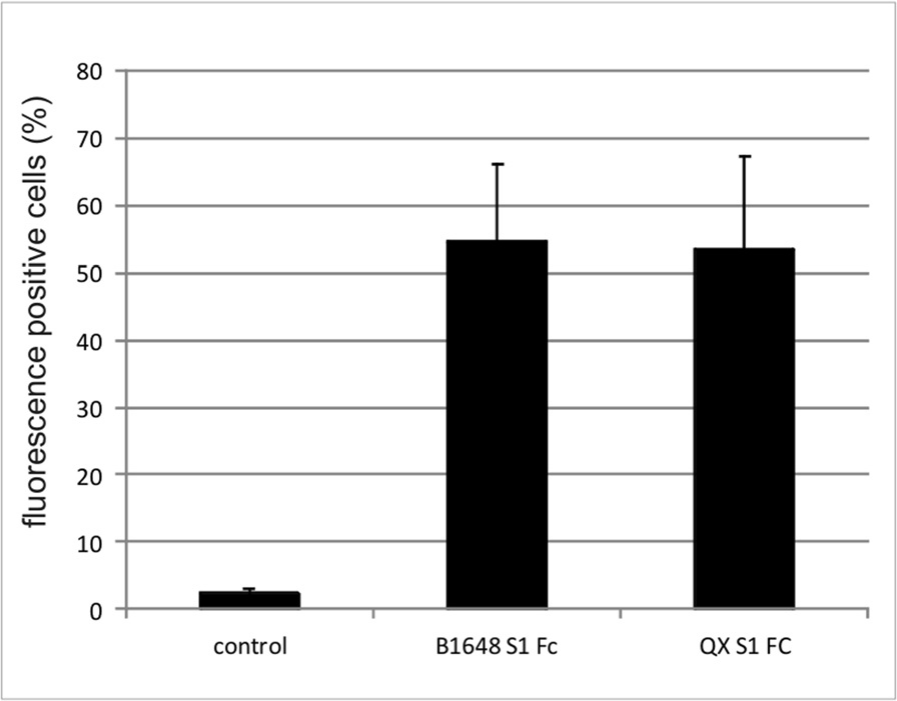
**Flow cytometric analysis of soluble S1 proteins of the strains QX and B1648 on isolated chicken oviduct epithelial cells.** Differentiated, isolated chicken oviduct epithelial cells were incubated with soluble spike proteins to detect the percentage of cells positive for bound spikes. No significant differences in the binding efficiency between S1 spike proteins of QX and B1648 was observed. Standard deviation is calculated from three separate experiments with each group in duplicates. Flow cytometric analysis of soluble S1 proteins of the strains QX and B1648 on isolated chicken oviduct epithelial cells.

To get quantitative data on the binding properties of the two spike proteins FACS analysis was performed with primary isolated oviduct epithelial cells that retained their differentiated status, which could be estimated by analysing the ciliary activity of the isolated epithelial cells under the light microscope. The isolated oviduct epithelial cells were incubated with the soluble spike proteins and analysed by flow cytometric analysis for the percentage of cells with bound spike proteins. No significant differences in the binding efficiency between S1 spike proteins of QX and B1648 was observed (Figure 7).

## Discussion

Analysis of IBV infections under in vitro conditions is difficult. Most strains do not replicate in permanent cell cultures. We could demonstrate in former studies, that primary cell culture systems of the respiratory tract are suitable tools to investigate and compare different IBV strains [11,12]. As IBV infection of the reproductive tract is one major economic problem in laying flocks, we developed in the present study an organ culture system for the chicken oviduct. This system allows us to study infections of differentiated epithelial cells under controlled in vitro conditions. Isolation and cultivation of chicken oviduct epithelial cells has been described by others [13,14], and also the preparation of oviduct explants -displaying the epithelial cells in their original settings as in vivo*-* has been shown to be a useful method to analyse IBV infections [15-17]. Our system has the advantage that it displays the immature organ, which differs in the epithelial composition from the mature organ [18]. In contrast to other studies we did not use hormones to achieve an artificial maturation of the oviduct. As an infection in very young immature layers can lead to the so called false layer syndrome, our immature epithelial cells might reflect this situation better that cultures from hormone treated chickens.

Binding to the host cell is the first important step in a virus life cycle. It is well accepted, that the affinity to certain receptors is a crucial point in determining the organ and/or species tropism. The IBV strains of different serotypes show a pronounced variation in the amino acid sequence (about 20-25%) of their S1 proteins (reviewed in [1]). As the spike protein is the major antigen it is not surprising, that viral evolution in large poultry flocks drives the emergence of new variants with modified spike genes. The spike protein is the attachment protein and the binding domain is located within in S1 subunit. Due to the high variance in the S1 sequences of different IBV strains- we asked the question if theses sequence variations are related with different binding properties of the spike protein and therefore may give some explanations for the different organ tropism of the strains. In former studies we already observed, that the binding to alpha 2,3-linked sialic acids expressed on host cells is important for initiating an infection. In the present study, we analysed the sensitivity of primary chicken oviduct epithelial cells to infection of the strains B1648 and QX. The observed differences correspond to results in former studies. The QX strain shows also in other primary cell cultures systems a high infectivity rate. In the bronchial epithelium it infected the cells with a higher efficiency than the IBV strains Beaudette, 4/91 and Italy02 [12]. In the oviduct explant cultures infection with the nephropathogenic B1648 strain was only inconstantly observed in the infundibulum part. The results obtained with in vitro explant cultures reflect the situation in vivo to some extent. While QX has been reported to affect the chicken oviduct during infection, with the B1648 strain no reproductive disorders are described. When we compared the binding of the two S1 proteins from both strains on the oviduct cells by using soluble S1 subunits, we found both spike proteins binding to the oviduct epithelial cells of all parts of the oviduct, indicating that the attachment might not be the limiting factor for B1648 to infect oviduct epithelial cells. This binding of the spike proteins was with both strains and in all segments of the oviduct dependent on sialylated glycans expressed on the cells. A treatment with a neuraminidase to cleave off sialic acids resulted in clearly reduced binding of the soluble spike proteins. Lectin staining with MAAII of the epithelial cells of different segments revealed an expression of alpha 2,3 linked sialic acids, the receptor determinant of IBV, in all parts. However, the varying binding of MAAI and SNA to the oviduct segments shows that there are differences in the glycosylation of the epithelial cells in the different segments of the oviduct.

Our results indicate that the difference in tissue tropism towards the chicken oviduct of the analysed strains seems not to be related to binding to the receptor determinant sialic acid on the cell surface. The binding properties of the spike proteins of both strains allow binding and therefore the critical first step to virus replication in oviduct epithelial cells [19]. As virus particles of the QX and the B1648 strain are also able to bind to oviduct epithelial cells, the observed binding of the soluble spike proteins resembles the binding of virions. Therefore the soluble spike proteins are a valuable tool to analyse binding properties of IBV strains.

The different sensitivity of the cells to infection with the two analysed strains asks for a different explanation. Possibly the binding to certain unknown main or co-receptors, allowing the viral uptake via endocytosis, is mediated only by the QX spike and not by the B1648 spike. It has been demonstrated for several alpha and beta coronaviruses as well as for the IBV Beaudette strain that an additional binding property to heparan sulphate may help to broaden the tropism to new hosts [20-22]. So it could be that we observe only the first (initial) binding to the sialic acids with the soluble spike constructs, and could not detect or distinguish any later binding step necessary for infection. This binding may also be conformation dependent and may therefore depend on correctly folded spike proteins or the presence of the S2 subunit. Promkuntod et al. [19] have shown, that the presence of the S2 subunit in soluble spike protein constructs could improve the binding. Another explanation could be that a post binding step in the virus life cycle e.g. the fusion process in the oviduct epithelial cells is not mediated by all spike proteins to the same efficiency. The presence of certain proteases to activate the fusionability of the spike proteins may play a role. As IBV strains differ in their sequences within the cleavage sites of their spike proteins this could have an impact on the proteolytic activation by host proteases.

Our primary cell cultures are a highly valuable tool to investigate IBV infections, with some limitations. Infection in the chicken is a much more complex situation. Ex vivo experiments cannot include influences of immune mechanisms and if the virus could reach the oviduct at all. There is little information about the way an IBV infection spreads in the chicken. It cannot be ruled out that a different -strain specific- efficiency to reach the oviduct may influence the organ tropism. However, our explant culture system allows us to analyse the sensitivity of the epithelial cells to viruses in a reproducible way and shows that these differences may impact the organ tropism and subsequently the pathotype of a certain IBV strain.

Future experiments have to further investigate the characteristics and mechanisms allowing successful IBV infection of oviduct epithelial cells.

## Competing interests

The authors declare to have no competing interests.

## Authors’ contributions

AKM carried out the experiments and wrote the manuscript. MH participated in the FACS analysis and molecular cloning. SAE participated in the explant and cryosection preparation. SR and GH participated in the design of the study. CW designed the study and wrote the manuscript. All authors read and approved the final manuscript.

## Authors’ information

This work was performed by AKM in partial fulfillment of the requirements of a PhD degree from the University of Veterinary Medicine Hannover. This work was supported by a grant to AKM from the Hannover Graduate School for Veterinary Pathobiology, Neuroinfectiology, and Translational Medicine (HGNI) donated by Boehringer Ingelheim.

## Supplementary Material

Additional file 1 **S1 Sequences of QX and B1648 strains.** The S1 cDNA sequences of the used IBV strains QX and B1648 are shown in FASTA format. Click here for file
