# Supplementary material for: Differences in the tissue tropism to chicken oviduct epithelial cells between avian coronavirus IBV strains QX and B1648 are not related to the sialic acid binding properties of their spike proteins
Source: Vet Res. 2014 Jun 14;45(1):67. doi: 10.1186/1297-9716-45-67 (PMC4076756; doi:10.1186/1297-9716-45-67)
Supplement: Additional file 1 — S1 Sequences of QX and B1648 strains. The S1 cDNA sequences of the used IBV strains QX and B1648 are shown in FASTA format. [file 1297-9716-45-67-S1.pdf]

>QX S1

atgttggtgaagtcactgtttttagtgaccattttgtgtgcactatgtagtgcaaatttg

tttgattccgataataattatgtgtactactaccaaagtgcttttagaccgccaaatggg

tggcatctacaaggaggtgcttatgcagtagtcaattctactaattatactaataatgcc

ggttctgcacaagggtgcactgttggtgttattaaggatgtttataatcaaagtgtggct

tccatagctatgacagcacctcttcagggtatggcttggtctaaggcacaattctgtagt

gcacactgtaacttttctgaaattacagtttttgtcacacattgttatagtagtggtagt

gggtcttgtcctataacaggcatgattccaagtggtcatattcgtatttctgcaatgaaa

aatggtttttttattttataatttaacagttagcgtatctaaataccctaattttaaatct

tttcaatgtgttaacaacttcacatctgtttatttaaatggtgatcttgtttttacttcc

aacaaaactactgatgttacgtcagcaggtgtgtattttaaagcaggtggacctgtaaat

tataatattatgaaagaatttaaggttcttgcttactttgttaatggtacagcacaagat

gtaattttgtgcgacaattcccccaagggtttgctagcttgtcaatataatactggcaat

ttttcagatggcttttatccttttactaatagtactttggttagggaaaagttcatcgtc

tatcgcgaaagtagtgttaatactactctggcgttaactaatttcacttttattaatgaa

agtaatgcacagcctaatagtggtggtgttaatacttttcacttataccaaacacaaaca

gctcagagtggttattataattttaatttgtcatttctgagtcagtttgtgtataaggca

agtgattttatgtatgggtcctaccaccctcgttgtccttttaaaccagaaaccattaat

agtggtttatggtttaattccttgtcagtttctcttacttatggacccctacagggaggg

tgtaagcaatctgtttttagtggtaaggcaacgtgttgttacgcctactcttataacggc

ccaatggcatgtaaaggtgtttattcaggtgaattaagcacgaattttgaatgtggattg

ctggtttatgttactaagagtgatggctctcgtatacagactagaacagagcccttagta

ttaacgcaacacaattataataatattactttagataagtgtgttgcctataatatatat

ggcagagtaggccaaggttttattactaatgtgactgattctgctgttaattttagttat

ttagcagatggtgggttagctattttagatacgtcgggtgccatagatgtttttgttgta

cagggcatctatggtcttaattattacaaggttaatccttgtgaagatgttaatcaacaa

tttgtagtgtctggtggcaatatagttggcattcttacttctagaaatgaaacaggttct

gaacaggttgagaaccagttttatgttaagttaaccaatagctcacatcgtcgtaggcgt

tctattggccaaaat

>B1648 S1

atgttggtagtgcaactttcagtagtgactcttttgtttgcactatgtagtgctattttg

ttcaatgataattataattattactaccaaagtgccttcagaccaccctcagggtggcat

ttacatgggggtgcttatcaagtggtcaatgttactaatgaaaataataatgcaggttca

tcaccaacatgcactgcaggtgttatttattatagtaaaaattttactgcttcttctgta

gccatgactgcaccaccaccaggaatgtcatggtccacttcagaattttgtacggcccac

tgtaattttagtacatttacagtgttcgttacacattgttttaaaagcggtgcaggccaa

tgtcctttaactggtttaatacaacagggatatattcgtgtctctgctatgaaaacggaa

ggtgaatcatacctattttataatttaacattgccaacgactaaacatcctaagtttagg

tcgctacaatgtgttaataatcaaacatctgtgtatttaaatggtcatcttgtctttact

tctaatgagactttagatgttagtgcggcaggtgtttattttaaatctggtggacctata

acttataaagttatgagagaagttaaagcccttgcttattttgttaatggtactgcacat

gatgtaattttgtgtgacaattcaccaaggggtttgttagcatgccagtacaacactggt

aatttttcagatggattttatccttttactaattcttctttagttaaggaaaagtttatt

gtttatcgtgaaagtagtgtgaatactacattagagttaactaacttcacattttcaaat

caaagtaatgctacacccaatagtggtggtgttaataccttttcattatatcaaacacac

acagctcagagtggttattataattttaacttttcttttctgagtgggtttacgtataaa

ccatccgattttatgtatgggtcatatcacccacgttgtaattttagaccagagaatatt

aataatggcttatggtttaattcattaactgtgtcacttacttatggacctcttcaaggt

ggttgtaagcaatcggtttttagtaatagagcaacttgttgttatgcttattcttataat

ggacccatatcttgtaaaggtgtttattcaggtgaattaaaccaatattttgaatgtggt

ttgttggtttatgttactaagagcgatggctctcgcatacaaacggcaacagaaccaccc

atttttacggaaaattattataacaacattactttaggtaagtgtgttgagtataatata

tatggtagatttggtcaaggttttattactaatgtaactgattcagctgctaattttaat

tatttagcagatggtggcttagctattttagatacgtctggagccatagacatctttgtt

gttcaaggtgactatggtcttaattattataaggttaatccctgtgaagatgtaaatcag

cagtttgtagtctctggtggtaatatagtaggtgtccttacatcaattaatgaaactggt

tctcaatttgttgggaatcagttttatgttaaactcactaatagtaca
